# Post-dengue parkinsonism

**DOI:** 10.1186/1471-2334-13-179

**Published:** 2013-04-18

**Authors:** Shahrul Azmin, Ramesh Sahathevan, Zainudin Suehazlyn, Zhe Kang Law, Remli Rabani, Wan Yahya Nafisah, Hui Jan Tan, Mohamed Ibrahim Norlinah

**Affiliations:** 1 Department of Medicine, Universiti Kebangsaan Malaysia Medical Centre, Kuala Lumpur, Malaysia

**Keywords:** Parkinsonism, Dengue fever

## Abstract

**Background:**

Dengue is a common illness in the tropics. Equally common are neurological complications that stem from dengue infection. However, to date, parkinsonism following dengue has not been reported in medical literature.

**Case presentation:**

A previously well 18-year old man developed parkinsonism, in addition to other neurological symptoms following serologically confirmed dengue fever. Alternative etiologies were excluded by way of imaging and blood investigations.

**Conclusions:**

The authors detail the first reported case of parkinsonism complicating dengue fever. Keeping rare presentations of common illnesses in mind, it behoves clinicians to consider parkinsonism as a complication following dengue infection. This would prevent injudicious treatment with L-dopa and dopamine agonists. Immunosuppression with steroids has been shown to be helpful in certain cases.

## Background

Dengue virus is a mosquito-borne single positive-stranded RNA virus of the *Flaviviridae* group [1]. Other viruses belonging to this group include West Nile virus, Murray Valley virus and Japanese Encephalitis virus. Neurological symptoms arising from dengue virus infection occur by 3 different pathological process; direct neurotropic invasion, systemic complication and post-infectious immune mediation [2]. The post-infectious neurological symptoms can be delayed by up to 2 weeks after the onset of viral fever and commonly manifests as mononeuropathies, polyneuropathies and Guillian-Barre syndrome [3].

## Case presentation

An 18-year old man was admitted to our hospital with a 3-day history of fever, associated with myalgia, lethargy and headaches. He reported gum bleeding when he brushed his teeth that morning. He had chicken pox 2 months prior to his admission but otherwise did not have any past medical history of note. He is a teetotaller and does not smoke cigarettes. On examination, his vital signs were as follows; temperature of 39.2°C, pulse rate of 110 beats per minute and blood pressure of 110/70 mmHg with evidence of postural hypotension. His respiratory rate was 24 per minute with oxygen saturation of 94% when inspiring room air. He had a macular confluent rash over the face, thorax and flexor surfaces but review of other systems was unremarkable on admission. Routine blood investigations were performed with the following results; hemoglobin (Hb) 15.8 g/dL, white blood count (WBC) 3.0 × 10^9^/L and a platelet count of 64 × 10^9^/L. Serum creatinine was 88 umol/L, serum sodium 129 mmol/L and potassium 3.7 mmol/L. Liver enzyme results were as follows: alanine transaminases 170 U/L, alkaline phosphatase 44U/L, bilirubin 14umol/L. Protein level was 67 g/L with albumin of 43 g/L. Dengue NS1 antigen was positive but dengue IgM and IgG antibodies were negative. He was diagnosed as acute dengue fever and treated with intravenous infusion of normal saline with strict management of his fluid intake and supportive therapy for his symptoms. The patient did not require any blood products during his illness.

He responded well to medical treatment and his symptoms improved by Day 3 of admission. On Day 6, the patient complained of unsteadiness, falling backwards when walking. This was associated with slurring of speech but he denied any difficulty swallowing food. He also complained of sudden weakness in the right arm, unable to lift it up above his head, which was associated with pain in the shoulder. His mother commented that he seemed slow in his movements and took a longer time to respond to questions.

A comprehensive neurological examination revealed an alert young man with a Glasgow Coma Scale of 15/15. His Mini-Mental State Examination (MMSE) was 30/30 although he was noted to be bradyphrenic and his speech was dysarthric. Cranial nerve examination revealed bilateral nystagmus but no opthalmoplegia. He had weakness of the right trapezius, right sternocleidomastoid muscle and his tongue was pushed to the right on protrusion. His gag reflex was intact. Examination of the remaining cranial nerves did not reveal any other abnormality. Examination of the limbs revealed weakness in the right deltoid muscle Medical Research Council (MRC) grade 3/5, right infraspinatus muscle MRC grade 3/5, with winging of the right scapula. There was no muscle atrophy. The reflexes were absent in the right biceps. He had bilateral dysmetria and intention tremor in the upper limbs. There was evidence of bilateral bradykinesia when performing finger and toe tapping. Proprioception and pin-prick sensation was normal. His gait was ataxic and broad based.

**Figure 1:**
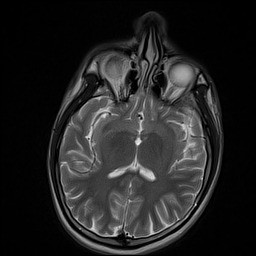
Axial brain MRI T2 sequence.

**Figure 2:**
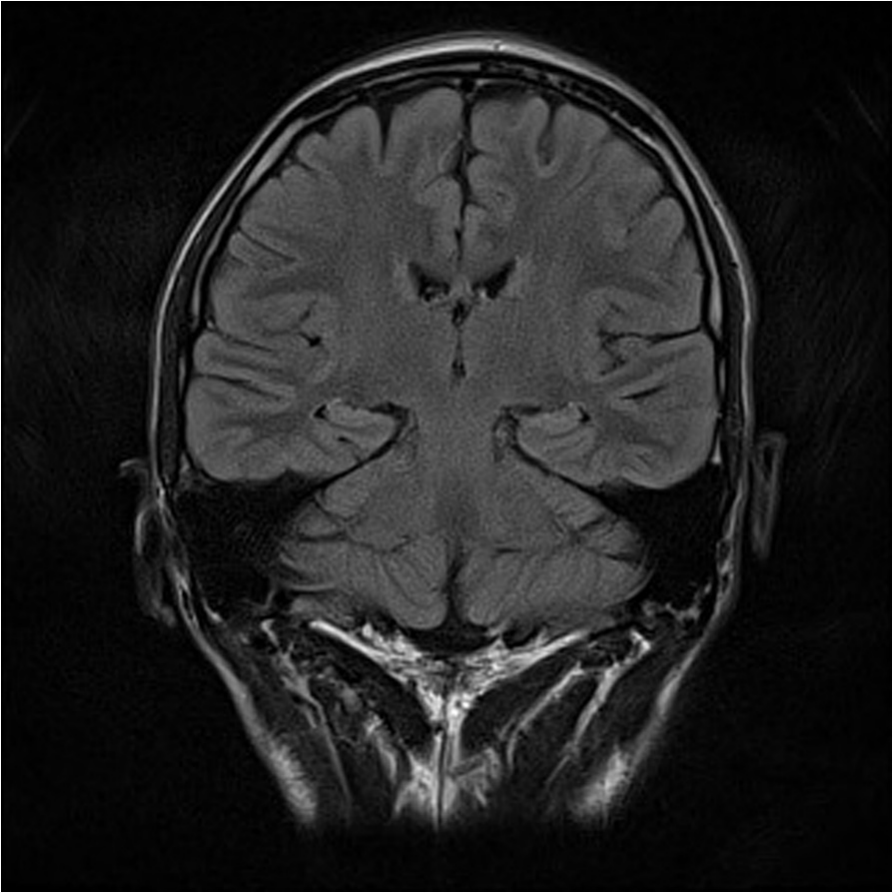
Coronal brain MRI with FLAIR sequence.

**Figure 3:**
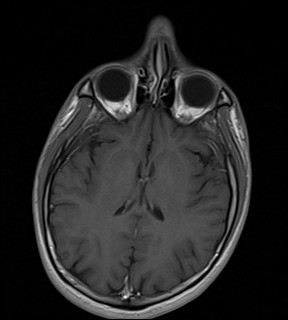
Gadolinium–enhanced axial brain MRI with T1 sequence.

Additional laboratory investigations were requested. A repeat IgM and IgG for dengue antibodies were positive at 1 week post admission. Serum B12 and serum folate levels were normal. ESR was elevated at 78 mm/hr. Anti-nuclear antibodies were negative, C3 and C4 levels were within normal limits. A cerebrospinal fluid (CSF) analysis showed no white blood cell or red blood cells, total protein of 557 mg/L and glucose of 2.5 mmol/L with corresponding blood glucose of 5.4 mmol/L. Cerebrospinal fluid culture for dengue virus and herpes simplex virus were negative. He was screened for viral hepatitis; Hepatitis A IgM, HBsAg and Anti-HCV were all non-reactive. Similarly, HIV antigen and antibody were also negative. A gadolinium-enhanced MRI scan of the brain with FLAIR sequence was requested and did not reveal any focal pathology (Figures 1, 2 and 3). A nerve conduction study was performed and the findings were unremarkable.

A diagnosis of parkinsonism with multiple cranial neuropathies, cerebellar ataxia and brachial plexopathy secondary to dengue fever was made. He was given intravenous methylprednisolone 500 mg OD for 3 days.

At 1-month review in clinic, his symptoms of parkinsonism and cerebellar ataxia had resolved but weakness in the right deltoid and infraspinatus muscles remained with marked muscle atrophy. The nerve conduction study was repeated and the findings were normal. However, electromyogram (EMG) revealed chronic denervation changes in the right deltoid and right trapezius muscle, consistent with right brachial plexopathy.

## Discussion

This case highlights 2 interesting points. Firstly, the rare complication of parkinsonism following dengue virus infection. Secondly, the extensive post-infectious neurological manifestations in a previously well young man with no history of chronic illness. We aim to address these issues in the next few paragraphs in turn.

The family *Flaviviridae* has been known to cause muscle weakness. Poliomyelitis-like weakness has been associated with Japanese encephalitis virus infection and West Nile virus infection [4,5]. To our knowledge, our patient is only the second reported case of dengue-fever associated brachial plexopathy [6].

Acute cerebellar ataxia is quite common following a viral infection; Varicella Zoster virus, Mumps virus and Epstein-Barr virus being the common culprits [7]. Cerebellar ataxia following dengue fever is less commonly reported [8].

Virus as the etiological agent for parkinsonism is not a novel concept. Von Economo first described seven patients presenting with parkinsonism, somnolence and diplopia; almost all had at least one cranial nerve involvement [9]. Although now a topic of much debate, initially the trigger was thought to be the influenza virus.

We feel that our patient developed features of parkinsonism as a post-infectious immune-mediated consequence of dengue fever. NS1 antigen has been shown to have a 93.4% sensitivity rate and 100% specificity rate in diagnosing acute dengue fever [10]. The diagnosis was further confirmed by the subsequent positive IgM and IgG for dengue antibodies in the serum. An acute parkinsonian syndrome has been described following Japanese Encephalitis virus and West Nile virus infection, but to our knowledge, our patient represents the first case of acute parkinsonian syndrome afflicting a patient following a dengue infection [11,12].

The absence of the dengue virus in the cerebrospinal fluid of our patient highlights an important point, and suggests that the mechanism by which the virus is causing the symptoms is not via a direct neurotropic effect [13]. There have been previous reports of viral encephalitis with no evidence of the virus apart from the presence of oligoclonal bands in the cerebrospinal fluid, suggesting an immune-mediated mechanism [14,15]. We did not test for oligoclonal bands but there was evidence of pleocytosis in the cerebrospinal fluid of our patient.

The absence of any abnormal findings on brain MRI scan further reinforces this hypothesis. Cranial neuropathy arising from post-infectious immune mediated dengue fever with normal brain MRI findings have been previously reported [16].

Although the phenomenology of viral parkinsonism may share similar characteristics with idiopathic Parkinson’s disease, it is unlikely that the pathophysiology is due to abnormal Lewy body and neurofibrillary tangle deposition in brain tissue [17]. The usual anti-parkinson’s medication such as levodopa and dopamine agonist may not be as effective. Indeed, as our case demonstrates, there is a role for immunosuppression with methylprednisolone in selected patients.

Our patient developed quite extensive neurological symptoms involving the extra-pyramidal system, cranial and peripheral nerves. The patient was an otherwise fit young man with no history of chronic illness, apart from having chicken pox 3 months earlier. Although remote, the possibility of varicella zoster virus playing a role in the subsequent neurological complication of dengue fever should not be dismissed out of hand.

One-way to explain this unusual phenomenon is the ‘double-hit’ hypothesis. It is known that influenza virus has the capability to prime the innate CNS immune system [18]. If varicella zoster virus has the same capability in up-regulating the CNS immune system, a ‘second-hit’ in the form of dengue fever may explain why our patient was afflicted with such diverse neurological manifestation, namely, parkinsonism, multiple cranial neuropathies, cerebellar ataxia and plexopathy.

## Conclusions

This is the first reported case of parkinsonism in a patient who had dengue fever. Furthermore, our patient developed an extensive variety of neurological manifestations in addition to parkinsonism. The question of whether varicella zoster and dengue virus co-infection confers any prognostic significance on post-infectious neurological manifestations needs to be investigated further. Finally, in terms of treatment, immunosuppression with intravenous methylprednisolone may be useful in selected cases in aborting neurological progression and hastening recovery.

### Consent

Written informed consent was obtained from the patient for publication for this case report and any accompanying images. A copy of the written consent is available for review by the editor of this journal.

## Competing interest

The authors declare that they have no competing interests.

## Authors’ contributions

SA, ZS and RS have been involved in the drafting of the manuscript. ZKL, RR, WYN, HJT and MIN have been involved in critically revising the manuscript for important intellectual content. All authors read and approved the final manuscript.

## Pre-publication history

The pre-publication history for this paper can be accessed here:

http://www.biomedcentral.com/1471-2334/13/179/prepub
